# Gender variations in the relationship between social capital and mental health outcomes among the Indigenous populations of Canada

**DOI:** 10.1186/s12939-019-1028-9

**Published:** 2019-08-14

**Authors:** Alexander Levesque, Amélie Quesnel-Vallée

**Affiliations:** 1 Department of Sociology, McGill University, Montreal, Quebec H3A 2T7 Canada; 2 Canada Research Chair in Policies and Health Inequalities, Montreal, Quebec Canada; 3 McGill Observatory in Health and Social Services Reforms, Montreal, Quebec H3A 0E6 Canada; 4 Present Address: Schulich School of Medicine and Dentistry, University of Western Ontario, London, Ontario N6A 5C1 Canada

**Keywords:** Gender, Indigenous health, Social capital, Self-rated mental health, Heavy episodic drinking

## Abstract

**Background:**

In this paper we examine the relationship between social capital and two mental health outcomes—self-rated mental health (SRMH) and heavy episodic drinking (HED)—among the Indigenous populations of Canada. We operationalize a unique definition of social capital from Indigenous specific sources that allows for an analysis of the importance of access to Indigenous networks and communities. We also examine gender variations in the relationship between social capital and the two outcomes, as there is a noticeable lack of research examining the influence of gender in the recent literature on the mental health of Indigenous populations in Canada.

**Methods:**

Using data from the 2012 cycle of the Aboriginal Peoples Survey, logistic regression models were estimated to assess if gender was a significant predictor of either SRMH or HED among the entire Indigenous sample. The sample was then stratified by gender and the relationship between two social capital variables—one general and one indigenous-specific—and each mental health outcome was assessed separately among male and female respondents. All analyses were also further stratified into specific Indigenous groups—First Nations, Métis, or Inuit—to account for the unique cultures, histories, and socioeconomic positions of the three populations.

**Results:**

Female respondents were more likely to report fair or poor SRMH in the total sample as well as the First Nations and Métis subsamples (OR = 1.48, CI = 1.14–1.91; OR = 1.63, CI = 1.12–2.36; OR = 1.44, CI = 1.01–2.05 respectively). However, female respondents were less likely than males to engage in weekly HED in all three of the same populations (OR = 0.43, CI = 0.35–0.54, all respondents; OR = 0.42, CI = 0.31–0.58, First nations; OR = 0.39, CI = 0.27–0.56, Métis). Social capital from sources specific to Indigenous communities was associated with lower odds of weekly HED, but only among Indigenous men. Meanwhile the strength of family ties was associated with lower odds of reporting fair/poor SRMH among both Indigenous men and women. However, these results vary in strength and significance among the different Indigenous populations of Canada.

**Conclusions:**

The results of this paper address a critical gap in the literature on gender differences in SRMH and HED among the Indigenous populations of Canada, and reveal gendered variations in the relationship between social capital and SRMH and HED. These findings support further investigation into the role that social capital and particularly Indigenous-specific forms of social capital may play as a determinant of health. This research could contribute to future mental health initiatives aimed at strengthening the social capital of Indigenous populations and promoting resilient Indigenous communities with strong social connections.

## Background

In research on the mental health of Indigenous populations in Canada there is a growing trend towards recognizing the important role that Indigenous histories and cultures play as determinants of health. For example, recent research has examined the relationship between speaking an Indigenous language or participating in traditional Indigenous activities and mental health [1, 2]. There is also increased recognition of the ongoing impact of colonialism on the intergenerational transmission of Indigenous histories and cultures, and the resulting effects on the health and wellbeing of Indigenous communities. Colonial instruments, such as the residential school system and laws governing membership in Indigenous communities, continue to have gender-specific impacts on access to Indigenous networks and communities that are critical for many Indigenous people’s wellbeing [3]. This paper seeks to contribute to this growing body of literature by using the 2012 cycle of the Aboriginal Peoples Survey (APS) to examine the relationship between social capital from Indigenous-specific sources and two mental health outcomes—self-rated mental health (SRMH) and heavy episodic drinking (HED).

SRMH and HED are both potential indicators of a persons overall mental health, and are helpful when studying populations that, like many Indigenous populations, face significant systemic barriers in accessing formal mental health services [4]. In this case we have elected to study both outcomes to help account for the different manifestations of mental health among Indigenous men and women. Based on previous literature, we predict that Indigenous women will on average report worse SRMH and Indigenous men will report higher incidences of HED [2, 5]. Studying both outcomes allows us to assess the relationship between social capital and mental health outcomes that are both more common and less common among each gender. Examining the influence of gender on the relationship between social capital and mental health also helps acknowledge and account for the significant gender-specific in experiences with colonialism present in the Indigenous populations of Canada [6, 7].

### The significance of self-rated mental health and heavy episodic drinking

SRMH and HED are useful tools for broadly examining mental health, since they are both indicative of a persons’ overall mental health, but the presence of poor SRMH or HED does not necessarily conclude a mental health disorder. This is especially helpful when studying Indigenous populations that often face systemic barriers in accessing health services, such as discrimination or geographically inaccessible services, which may impact rates of medically diagnosed mental illness [3, 8].

SRMH measures offers a more holistic approach to studying mental health, which is especially important in research on Indigenous populations, as several Indigenous scholars stress the importance of holistic health to many Indigenous peoples [9–12]. Furthermore, poor/fair SRMH ratings are significantly more likely among those reporting a diagnosed mental disorder and those meeting the criteria for past-month depression [9], which makes the measure a useful proxy for mental health disorders that may be under diagnosed among Indigenous populations due to systemic barriers. Low SRMH is also linked to other negative mental health outcomes, including distress and suicidal ideation, that are currently prevalent in serious numbers in the Indigenous populations of Canada, further supporting the measures’ significance among the Indigenous population [1, 10, 11]. Overall, SRMH allows for a more holistic assessment of mental wellbeing, without requiring respondents to have a medically diagnosed disorder.

HED—typically operationalized as five or more drinks on one occasion—is also a potentially useful tool for examining mental health among the Indigenous population, as it is associated with many of the same negative mental health outcomes that are prevalent in some Indigenous communities. For example, excessive alcohol use correlates with rates of suicidal ideation and attempts, as well as self-reported feelings of depression and anxiety [13–16]. HED is also generally more prevalent than diagnosed mental health disorders [13–16], and can therefore serve as a useful indication of overall mental wellbeing in contexts where systemic barriers may lead to an under diagnosis of mental health disorders. Overall, both SRMH and HED can offer insight into wellbeing and overall mental health, albeit in different ways.

### Social capital as a predictor of mental health outcomes

Social capital can be broadly defined as relationships, networks, and norms that individuals can access to serve their interests [17]. Previous research on a variety of different populations and contexts has linked social capital variables—community participation, trust, cohesion, family connections, etc.—to self-rated mental health, psychological wellbeing, and other mental health outcomes [18–21]. Several studies have also examined the link between social capital and alcohol consumption, although many of these studies focused exclusively on adolescent populations and those that examined adult populations revealed conflicting results [22–27]. Overall, there is substantial evidence that social capital can be a salient predictor of mental health outcomes, including alcohol consumption, in a variety of contexts.

Although the relationship between social capital and mental health outcomes has not been thoroughly studied among the Indigenous populations of Canada, community integration is known to be integral to understandings of wellbeing among many of these populations. Traditional Indigenous conceptualizations of health are often holistic, encompassing not only physical wellbeing but also mental, emotional, and spiritual wellbeing [12]. Connections to the land, the use of language, cultural food practices, and the strength of interpersonal relationships are all important components of many Indigenous people’s understanding of health [12]. Throughout the last few centuries, colonialism in Canada has contributed to many of the aforementioned mental health problems that Indigenous communities face today through directly undermining these communities’ access to Indigenous networks, cultural norms, and relationships [12, 28]. For example, colonial instruments such as forced relocation and residential schooling created physical barriers separating Indigenous individuals from communities, while also decreasing the transmission of Indigenous cultural norms and practices, such as the use of Indigenous languages, cultural food preparation, or traditional healing practices, that were central to many Indigenous people’s wellbeing [12, 28]. Taken together, the importance of community integration in many Indigenous people’s conceptualization of health, and the continued degradation of this integration by colonialism, illustrate the potential role of social capital as a predictor of mental health outcomes among Indigenous populations.

In this paper we adopt an operationalization of social capital that allows for an analysis of Indigenous-specific social capital—that is, social capital coming from Indigenous relationships, networks, and norms. Proxies for Indigenous-specific social capital include factors like participation in traditional Indigenous activities and involvement in Indigenous communities [17]. Currently these proxies are often included in models individually—e.g. examining if speaking an Indigenous language predicts heavy drinking [2]. This can lead to conflicting results where participation in Indigenous communities and activities sometimes appears to be a protective factor against disease and other times not [1, 2, 29]. Social capital offers a different way of conceptualizing the relationship between these proxies and mental health outcomes, which could overcome the weaknesses of examining them individually. Therefore, we adopt here a holistic view to look at overall levels of immersion in Indigenous communities and networks—i.e. social capital—as opposed to whether or not participation in a single traditional Indigenous activity is correlated with a single outcome.

### Gender variations in health among indigenous populations

Gender is increasingly recognized as a key social determinant of health, as there are significant gender differences in many predictors of health [30, 31]. For example, women are on average more socioeconomically disadvantaged and face more job precarity than men [31, 32]. However, young men typically engage in more risky behaviors than women, such as recreational drug use, which can negatively impact their health later in life [31]. Despite this increased recognition of gender as an important determinant of health, in the recent literature on the mental health of Indigenous people in Canada there is a noticeable lack of papers examining the influence of gender [1]. This literature gap is surprising considering significant evidence that Indigenous peoples’ experiences with colonialism can be extremely gendered [3, 33].

Colonial instruments were often specifically designed to affect Indigenous men and women in different, unique ways [3, 6]. For example, until 1985 under Canadian law any Indigenous women who married a non-Indigenous man lost the right to membership in Indigenous communities—the same was not true for Indigenous men who married non-Indigenous women [3]. This had the effect of directly severing community and cultural ties for many Indigenous women and their descendants, ties that are critical for many Indigenous people’s wellbeing [3, 12]. Indigenous men, meanwhile, have been uniquely affected by the absence of fathering in many Indigenous communities, attributable in part to the legacy of the residential school system [34]. This absence has in turn contributed to the increased prevalence of mental health problems in these same communities [34]. The evidence on gendered experiences with colonialism and health outcomes among Indigenous populations in Canada suggests that gender may be strongly related to both social capital and the two mental health outcomes. Therefore, a significant portion of this paper focuses on examining gender variations in the relationship between social capital and the mental health outcomes of interest.

A recent review of the literature on gender and SRMH seems to suggest that women are more likely to report worse SRMH, although several papers report no difference between men and women [5]. There is also no identifiable research specifically examining gender differences in SRMH among the Indigenous populations of Canada. Furthermore, although research on HED among the general Canadian population consistently suggests that men engage in more HED than women, research on HED among Indigenous populations is inconclusive on the relationship between gender and HED [13, 35, 36]. A 2016 study by Ryan et al. on heavy drinking among First Nations and Métis in Canada using the Aboriginal Peoples Survey (APS) found that men had significantly higher rates of heavy drinking [2]. As we are using the same survey as Ryan et al., we predict similar results, although we also hope to expand on their findings by examining the First Nations, Métis, and Inuit populations separately.

The First Nations, Métis, and Inuit populations have distinct cultures and histories, and have had very different experiences with colonialism. For example, Métis, and in particular Métis women, were uniquely targeted by government legislation and treaties that sought to erase the Indigenous identity of Métis communities [34]. Despite these unique historical and cultural experiences, and the resulting unique demographics of each population [37], few papers on the mental health of Indigenous people in Canada examine the three populations separately [1]. In this paper, we examine the Indigenous population as a whole as well as the First Nations, Métis, and Inuit populations separately, to address this gap in the literature and allow for the possibility of unique outcomes among the different populations due to each group’s distinct histories, cultures, and demographics.

This paper further expands on the work of Ryan et al.—and other prior research examining gender variations in health among Indigenous populations—through our analysis of social capital as a predictor of SRMH and HED. Given that the literature on Indigenous health frameworks suggests gender may be related to both mental health outcomes and access to social networks, we predict that Indigenous-specific social capital will be a better protector against outcomes that are more prevalent among a certain gender. Indigenous-specific social capital should therefore have a more significant protective effect against HED among Indigenous men, since we expect HED to be more prevalent among Indigenous men than Indigenous women. Similarly, Indigenous-specific social capital should have a stronger protective effect against fair/poor SRMH among Indigenous women, as we expect that fair/poor SRMH will be more prevalent among Indigenous women than Indigenous men.

## Methods

This study draws from the 2012 cycle of the Aboriginal Peoples Survey (APS), which surveyed the Indigenous population of Canada, 6 years of age and over, excluding those living on reserves or in certain First Nations communities. We further limited our analysis to respondents age 19 through 44. The lower limit signifies the age at which drinking is legal in all Canadian provinces and the upper limit was imposed by three of the selected social capital variables, which were only posed to respondents younger than 45 years old.

The APS was not fully designed in accordance with the vision of Indigenous health research set out by the Canadian Institute of Health Research: Institute of Indigenous Peoples’ Health, or the goals outlined in the Tri-Council Policy Statement for conducting appropriate research involving Indigenous populations in Canada [38, 39]. Cognizant of this limitation, we attempted to ground our research framework in Indigenous, culturally appropriate theories drawing from research produced by Indigenous scholars, and to approach this research from a decolonizing lens that emphasizes cultural determinants of wellness.

### The indigenous peoples of Canada

When completing the APS, respondents were asked which of the three distinct Indigenous groups in Canada they identify as: First Nations, Métis, or Inuit. *First Nations* refers generally to Indigenous groups, communities, and people who historically lived in North America*—*excluding the Inuit and Métis [40]. The *Inuit* historically inhabited the circumpolar regions of Canada, Russia, the U.S., and Greenland, and share a unique culture and history [40]. *Métis* refers generally to Indigenous people who are descendent from unions between Europeans and First Nations people, and therefore have a very distinct legal status in Canada [40]. These three Indigenous groups are also distinct in terms of current socioeconomic environments and health outcomes [1, 37]. In this paper we have separated the three populations in our analysis, in order to acknowledge these differences and address the lack of mental health literature that examines these populations separately [1]. Respondents who identified as belonging to more than one Indigenous group were excluded, because this group made up too small a portion of the overall sample to be analyzed separately.

### Self-rated mental health and heavy episodic drinking

SRMH was determined by asking respondents to rate their general mental health as excellent, very good, good, fair, or poor. Following the literature [5, 9, 10, 41], this variable was recoded as excellent/very-good/good (*reference category*) versus fair/poor in the two logistic regression models. The APS also asked respondents how often in the past 12 months they have consumed five or more drinks on one occasion. Respondents could choose from never, less than once a month, once a month, 2 to 3 times a month, once a week, and more than once a week. This variable was recoded as non-weekly (*reference category)* versus weekly HED. Evidence suggests that more frequent HED may be more associated with depressive symptoms, as well as problems with alcohol abuse and associated suicidal ideation/attempts [15, 16, 42]. As the focus of this analysis is on SRMH and HED as indicators of mental health, it made sense to utilize the highest possible frequency of HED as the outcome. Too few respondents indicated engaging in HED more than once a week, so focusing on weekly HED allowed for an appropriate cell size.

### Social capital specific to indigenous populations

The social capital variable was derived from six separate variables, following a common practice in the social capital literature of combining several measures into a single scale or index [18, 21, 22, 25]: speaking an Indigenous language, weekly exposure to an Indigenous language inside or outside the home, participation in traditionally Indigenous activities within the past 12 months (hunting, fishing, trapping, gathering of wild plants, making clothing/footwear, making arts or crafts), support for Indigenous culture in school, participation in Indigenous cultural activities after school, and time spent with elders after school. The last three variables refer specifically to the last year that the respondent was in school, and are helpful for capturing school-age exposure to institutional networks and norms that could provide social capital. Having engaged in four or more of these activities was categorized as “high levels of social capital” while three or less corresponded to “low levels of social capital” (*reference category*).

A typical measure of social capital aims to capture the degree to which someone has access to networks and relationships within their community [17]. While participation in traditional Indigenous activities may seem to fit well with measures of community participation found in the literature on social capital [18, 19], the significance of language use may not be as immediately clear. One of the primary targets of the assimilation policies—historically employed by Canada to undermine the strength of Indigenous communities—was the use of Indigenous languages [43]. The use of Indigenous language is an important part of Indigenous health frameworks, and a strong protective factor against negative mental health outcomes for some communities [12, 44]. Speaking or exposure to an Indigenous language, in the presence of other markers of social capital, could be important indicators of access to networks and relationships for some Indigenous populations. Responses to the specific activities within this list likely vary significantly between individual Indigenous groups—as Indigenous peoples in Canada are not homogenous and do not hold the same cultural practices—which also supports combining the social capital proxies into a single measure.

### Family ties in community

In addition to examining social capital arising from access to Indigenous networks and norms, we also included a variable corresponding to a more traditional definition of social capital—family ties in community. Respondents were asked to rank the strength of their ties to family members living within their community—but in a different household—on a scale from 1 to 5. For the regression models this variable was dichotomized as weak ties (*reference category)*—ratings 1–3 or no family in community—versus strong ties—ratings 4 and 5. To avoid confusion, in the rest of the paper this variable is referred to as *strength of family ties* while the variable examining social capital arising from Indigenous sources is referred to as *Indigenous-specific social capital*.

### Socioeconomic and demographic control variables

The following variables were controlled for in the regression models: age, education, income, and rural/urban residence. The income variable consisted of tertiles derived from a continuous income variable that allowed respondents to input their exact annual income from the preceding year, including negative values. These tertiles were derived separately for the two analytic samples. Education included three categories: no secondary degree, secondary degree or equivalent (including respondents with some post-secondary education but no degree), and post-secondary certificate/degree/diploma. Finally, urban and rural designations were based on whether or not the respondent lived in an area designated as a census metropolitan area (CMA).

### Statistical analysis

For both outcomes—HED and SRMH—three models were estimated. The first model tested if gender predicted the outcome, while adjusting for age, education, income, and urban/rural environment. This was done to verify and draw comparisons to previous research on gender differences in these two outcomes. We then divided the samples by gender, and ran logistic regressions that only examined the relationship between *social capital* and each outcome—without including *strength of family ties*—while controlling for the same factors. Finally, the third model tested the predictive power of *Indigenous specific social capital* and *strength of family ties in community* on fair/poor SRMH and weekly HED—again on male and female respondents separately. All models and descriptive statistics were estimated with the bootstrap weights provided by Statistics Canada.

We chose to test the predictive power of social capital on the outcome variables for male and female respondents separately, rather than include an interaction term between social capital and gender, because such an interaction term would likely over-simplify the relationship between gender and the predictors/outcomes. The literature suggests that there are structural gender variations in the health outcomes and histories of Indigenous peoples in Canada—as well as in sociodemographic characteristics [5, 6, 34]—and a single interaction term would not account for this complexity.

## Results

### Self-rated mental health

Table 1 displays the number of respondents, by gender and Indigenous Identity, for the first sample populations, which was defined using the SRMH variable. Overall, this sample comprised 9890 respondents, 57% of which were women. Table 2 presents summary statistics from this first sample population. Overall, a higher proportion of female respondents (0.14) reported fair/poor SRMH than male respondents (0.09). A higher proportion of women also reported having obtained a post-secondary diploma (0.51 among females; 0.40 among males), while a higher proportion of men reporting a top-tertile income (0.42 among males; 0.28 among females).

**Table 1:** Sample Sizes for the Self-Rated Mental Health Population by Gender and Indigenous Identity

	Men	Women	Total
First Nations	2200	3000	5200 (53%)
Métis	1850	2300	4150 (42%)
Inuit	240	300	540 (5%)
Total	4290 (43%)	5600 (57%)	9890 (100%)

**Table 2:** Descriptive Statistics for Self-Rated Mental Health Population

Variable	Proportion of Men	Proportion of Women
Self-Rated Mental Health		
Good/Very Good/Excellent	0.91	0.86
Fair/Poor	0.09	0.14
Indigenous-Specific Social Capital		
Low-Levels	0.74	0.72
High-Levels	0.26	0.28
Family Ties in Community		
Weak	0.36	0.33
Strong	0.64	0.67
Rural/Urban		
Urban	0.74	0.76
Rural	0.26	0.24
Education		
No Diploma	0.21	0.16
Secondary School Diploma	0.39	0.33
Post-Secondary Diploma	0.40	0.51
Income Tertile		
Less than $13,700	0.28	0.32
$13,700–$34,900	0.30	0.40
More than $34,900	0.42	0.28
Identity		
First Nations	0.51	0.54
Métis	0.43	0.41
Inuit	0.06	0.05
Mean Age	31	32

Table 3 displays the results from the multivariate logistic regression of fair/poor SRMH on gender and the control variables. Gender was significantly associated with reporting fair/poor SRMH in all subpopulations, except among Inuit respondents where the relationship was of a similar magnitude but not significant. Female respondents were more likely to report fair/poor self-rated mental health—as opposed to good, very good, or excellent—in the overall, First Nations, and Métis populations (OR = 1.48, CI = 1.14–1.91; OR = 1.63, CI = 1.12–2.36; OR = 1.44, CI = 1.01–2.05 respectively). Age was significantly associated with SRMH in the overall (OR = 1.03, CI = 1.02–1.05) and First Nations (OR = 1.04, CI = 1.01–1.06) populations, with each one-year increase in age associated with higher odds of reporting fair/poor SRMH. Being in the top income tertile lowered the odds of reporting fair/poor SRMH in the total (OR = 0.50, CI = 0.34–0.73), Métis (OR = 0.45, CI = 0.27–0.75), and Inuit (OR = 0.28, CI = 0.13–0.62) populations. Living in a rural location—i.e. outside of a CMA—was significantly associated with lower odds of reporting fair/poor SRMH among the general sample population (OR = 0.56, CI = 0.40–0.79) and Métis subsample (OR = 0.55, CI = 0.36–0.83).

**Table 3:** Logistic Regression for self-rated mental health regressed on gender—separated by Indigenous identity

Variable	Odds Ratio (Confidence Interval)			
	Total Population	First Nations Population	Métis Population	Inuit Population
Gender				
Male	–	–	–	–
Female	1.56 (1.22–2.00)*	1.63 (1.13–2.34)*	1.49 (1.05–2.11*)	1.36 (0.80–2.29)
Age	1.03 (1.02–1.05)*	1.04 (1.01–1.06)*	1.03 (1.00–1.05)	1.03 (0.99–1.06)
Income Tertile				
Less than $14,000	–	–	–	–
$14,000–$35,700	0.83 (0.63–1.09)	0.90 (0.61–1.33)	0.76 (0.51–1.12)	0.57 (0.29–1.11)
More than $35,700	0.50 (0.34–0.73)*	0.56 (0.31–1.00)	0.45 (0.27–0.75)*	0.28 (0.13–0.62)*
Rural/Urban				
Urban	–	–	–	–
Rural	0.56 (0.40–0.79)*	0.60 (0.32–1.13)	0.55 (0.36–0.83)*	0.54 (0.24–1.20)
Education				
No Diploma	–	–	–	–
Secondary School Diploma	0.55 (0.39–0.77)*	0.41 (0.25–0.68)*	0.84 (0.54–1.30)	0.88 (0.46–1.66)
Post-Secondary Diploma	0.37 (0.27–0.51)*	0.28 (0.18–0.44)*	0.55 (0.35–0.86)*	0.76 (0.35–1.65)

95% confidence intervals; *p < 0.05

Table 4 displays the results of the regression of fair/poor self-rated mental health on Indigenous-specific social capital and the strength of family ties in the community, controlling for the sociodemographic variables. Although we also ran a regression of fair/poor SRMH only on *social capital*—without *family ties*—introducing family ties had no effect on the relationship between social capital and SRMH—i.e. it was trivial in every population with or without including *family ties*. Among the sample population as a whole, Indigenous-specific social capital was not significantly associated with the odds of reporting fair/poor SRMH among males or females. However, both male and female respondents with strong family ties in their community were significantly less likely to report fair/poor SRMH than respondents with weak family ties (OR = 0.43, CI = 0.28–0.65; OR = 0.49, CI = 0.36–0.67 respectively). The same relationship held true among First Nations respondents (OR = 0.36, CI = 0.19–0.66 for male respondents; OR = 0.59, CI = 0.38–0.91 for female respondents) and Métis respondents (OR = 0.50, CI = 0.30–0.85 for male respondents; OR = 0.35, CI = 0.22–0.56 for female respondents). The relationship was also significant among Inuit female respondents (OR = 0.46, CI = 0.23–0.92), and was of a similar magnitude, although not significant, among Inuit male respondents (OR = 0.54, CI = 0.27–1.09).

**Table 4:** Logistic regression for fair/poor self-rated mental health regressed on social capital and family ties in community—separated by identity and gender

**Total Sample Population**		
	Odds Ratio for Males (Confidence Interval)	Odds Ratio for Women (Confidence Interval)
Indigenous-Specific Social Capital		
Low-Levels	–	–
High-Levels	0.85 (0.56–1.28)	1.13 (0.80–1.59)
Family Ties in Community		
Weak	–	–
Strong	0.43 (0.28–0.65)*	0.49 (0.36–0.67)*
**First Nations**		
	Odds Ratio for Males (Confidence Interval)	Odds Ratio for Women (Confidence Interval)
Indigenous-Specific Social Capital		
Low-Levels	–	–
High-Levels	0.75 (0.43–1.29)	1.04 (0.66–1.66)
Family Ties in Community		
Weak	–	–
Strong	0.36 (0.19–0.66)*	0.59 (0.38–0.91)*
**Métis**		
	Odds Ratio for Males (Confidence Interval)	Odds Ratio for Women (Confidence Interval)
Indigenous-Specific Social Capital		
Low-Levels	–	–
High-Levels	1.02 (0.53–1.96)	1.25 (0.66–2.35)
Family Ties in Community		
Weak	–	–
Strong	0.50 (0.30–0.85)*	0.35 (0.22–0.56)*
**Inuit**		
	Odds Ratio for Males (Confidence Interval)	Odds Ratio for Women (Confidence Interval)
Indigenous-Specific Social Capital		
Low-Levels	–	–
High-Levels	2.79 (0.91–8.57)	1.54 (0.59–3.97)
Family Ties in Community		
Weak	–	–
Strong	0.54 (0.27–1.09)	0.46 (0.23–0.92)*

95% confidence intervals; *p < 0.05; adjusting for age, education, income, and urban/rural location

### Heavy episodic drinking

Table 5 displays sample sizes and Table 6 displays summary statistics for the second sample population—derived using the HED variable. Overall, the entire sample comprised 8620 respondents, 54% of which were women. Among male respondents a higher proportion indicated that they engage in weekly HED (0.16) than among females (0.08), the opposite of the statistics on SRMH. The remaining statistics were very similar to those from the first sample: a higher proportion of male respondents were also in the top income tertile (42% of men; 28% of women), while a higher proportion of female respondents had obtained a post-secondary diploma (52% of women; 40% of men) and a smaller proportion had obtained no diploma (14% of women; 20% of men).

**Table 5:** Sample Sizes for the Heavy Episodic Drinking Population by Gender and Indigenous Identity

	Men	Women	Total
First Nations	1980	2480	4460 (52%)
Métis	1760	1980	3740 (43%)
Inuit	200	220	420 (5%)
Total	3940 (46%)	4680 (54%)	8620 (100%)

**Table 6:** Descriptive Statistics for Heavy Episodic Drinking Population

Variable	Proportion Among Men	Proportion Among Women
Weekly Heavy Episodic Drinking		
No	0.84	0.92
Yes	0.16	0.08
Indigenous-Specific Social Capital		
Low-Levels	0.75	0.73
High-Levels	0.25	0.27
Family Ties in Community		
Weak	0.36	0.32
Strong	0.64	0.68
Rural/Urban		
Urban	0.74	0.78
Rural	0.26	0.22
Education		
No Diploma	0.20	0.14
Secondary School Diploma	0.40	0.34
Post-Secondary Diploma	0.40	0.52
Income Tertile		
Less than $14,000	0.28	0.32
$14,000–$35,700	0.30	0.40
More than $35,700	0.42	0.28
Identity		
First Nations	0.50	0.53
Métis	0.45	0.42
Inuit	0.05	0.05
Mean Age	31	32

Table 7 contains the results from the multivariate logistic regression of weekly HED on gender and the control variables. Gender was significantly associated with weekly HED in all subsamples except for the Inuit population. Female respondents were less likely than males to engage in weekly HED in general (OR = 0.43, CI = 0.35–0.54), and this was also true for First Nations (OR = 0.42, CI = 0.31–0.58) and Métis respondents (OR = 0.39, CI = 0.27–0.56) as well. Of the control variables, having a post-secondary diploma was associated with lower odds of weekly HED, but only when examining the total (OR = 0.67, CI = 0.49–0.90) and Métis (OR = 0.56, CI = 0.36–0.86) populations. The direction and magnitude of both associations was the same among Inuit respondents, though they did not reach statistical significance.

**Table 7:** Logistic Regression for weekly heavy episodic drinking regressed on gender—separated by Indigenous identity

Variable	Odds Ratio (Confidence Interval)			
	Total Population	First Nations Population	Métis Population	Inuit Population
Gender				
Male	–	–	–	–
Female	0.43 (0.35–0.54)*	0.42 (0.31–0.58)*	0.39 (0.27–0.56)*	0.69 (0.42–1.13)
Age	0.99 (0.98–1.01)	0.99 (0.97–1.01)	1.00 (0.97–1.02)	0.98 (0.94–1.03)
Income Tertile				
Less than $14,000	–	–	–	–
$14,000–$35,700	1.02 (0.79–1.32)	1.13 (0.78–1.65)	0.88 (0.59–1.32)	1.08 (0.58–2.00)
More than $35,700	0.91 (0.66–1.25)	1.04 (0.65–1.68)	0.76 (0.49–1.19)	1.03 (0.51–2.07)
Rural/Urban				
Urban	–	–	–	–
Rural	0.94 (0.75–1.17)	0.81 (0.56–1.17)	1.14 (0.80–1.63)	0.52 (0.25–1.07)
Education				
No Diploma	–	–	–	–
Secondary School Diploma	0.87 (0.65–1.16)	0.94 (0.61–1.46)	0.77 (0.51–1.17)	1.06 (0.60–1.88)
Post-Secondary Diploma	0.67 (0.49–0.90)*	0.78 (0.50–1.21)	0.56 (0.36–0.86)*	0.58 (0.32–1.06)

95% confidence intervals; *p < 0.05

Table 8 displays the results from the regression of weekly HED on Indigenous-specific social capital and the strength of family ties in the community. While we also ran a model regressing weekly HED only on *Indigenous-specific social capital*—without *strength of family ties*—adding the *family ties* variable to the model only affected the significance of the relationship between the dependent and independent variables among Inuit males—and had no other effects. For this reason, the model was included in the Appendix (Table 9), but not in the body of the results section.

**Table 8:** Logistic regression for weekly heavy episodic drinking regressed on social capital and family ties in community—separated by identity and gender

**Total Sample Population**		
	Odds Ratio for Men (Confidence Interval)	Odds Ratio for Women (Confidence Interval)
Indigenous-Specific Social Capital		
Low-Levels	–	–
High-Levels	0.68 (0.51–0.91)*	0.91 (0.64–1.30)
Family Ties in Community		
Weak	–	–
Strong	1.03 (0.76–1.38)	0.76 (0.54–1.09)
**First Nations**		
	Odds Ratio for Men (Confidence Interval)	Odds Ratio for Women (Confidence Interval)
Indigenous-Specific Social Capital		
Low-Levels	–	–
High-Levels	0.53 (0.35–0.81)*	0.69 (0.41–1.16)
Family Ties in Community		
Weak	–	–
Strong	1.11 (0.72–1.70)	0.61 (0.38–0.97)*
**Métis**		
	Odds Ratio for Men (Confidence Interval)	Odds Ratio for Women (Confidence Interval)
Indigenous-Specific Social Capital		
Low-Levels	–	–
High-Levels	0.96 (0.60–1.56)	0.89 (0.47–1.70)
Family Ties in Community		
Weak	–	–
Strong	1.00 (0.66–1.53)	0.98 (0.52–1.83)
**Inuit**		
	Odds Ratio for men (Confidence Interval)	Odds Ratio for Women (Confidence Interval)
Indigenous-Specific Social Capital		
Low-Levels	–	–
High-Levels	0.36 (0.14–0.94)*	3.02 (1.10–8.36)*
Family Ties in Community		
Weak	–	–
Strong	0.98 (0.49–1.94)	1.35 (0.72–2.53)

95% confidence intervals; *p < 0.05; adjusting for age, education, income, and urban/rural location

The results from Table 8 suggest that male respondents with high levels of Indigenous-specific social capital—i.e. indicated the presence of four or more social capital variables—were 32% less likely to report weekly HED (CI = 0.51–0.91) than male respondents with low levels of social capital. However, a significant relationship between Indigenous-specific social capital and HED was not found among female respondents. Among First Nations respondents we found almost identical results to those found among all Indigenous respondents. High levels of Indigenous-specific social capital are only significantly associated with lower odds of weekly HED among men (OR = 0.53, CI = 0.35–0.81). However, female First Nations respondents with strong rather than weak family ties in the community were also less likely to engage in weekly HED (OR = 0.61, CI = 0.38–0.97)—this is not observed among all Indigenous respondents, Métis respondents, or Inuit respondents. While high levels of Indigenous-specific social capital were significantly associated with lower odds of weekly HED for both Indigenous and First Nations males, this relationship was not significant among Métis men.

Inuit men with high levels of Indigenous-specific social capital were also significantly less likely to report weekly HED (OR = 0.36, CI = 0.14–0.94). However, this relationship was not significant when the model was run without the *strength of family ties* variable (OR = 0.36, CI = 0.13–1.01, Table 9). While this suggests that Indigenous-specific social capital and the strength of family ties may be related among the Inuit population, the relatively small sample size of the Inuit population (5% of the male sample population) makes it difficult to emphasize this finding. Similarly, among Inuit women high levels of social capital was associated with much *higher* odds of reporting weekly HED (OR = 3.02, CI = 1.10–8.36). The cell size for this analysis exceeded the minimum requirements put forth by Statistics Canada, but any interpretation should be weighed against the small sample size and very large confidence interval.

## Discussion

### Relationship between gender and mental health outcomes

The results in this paper offer an important contribution to the overall debate on gender differences in SRMH scores. Ahmad et al.’s scoping review on SRMH found contradictory evidence on the relationship between gender and self-rated mental health—several papers concluded that women reported poorer SRMH, and others found no difference between men and women [4]. Among papers specifically examining Canadian populations the evidence on the relationship between gender and self-rated mental health is also mixed [1, 4, 41]. We found that First Nations and Métis women were significantly more likely to report fair/poor SRMH than male respondents. Future research could perhaps compare the strength of the relationship between gender and SRMH, between the Indigenous and general populations of Canada to see if gender is a more important predictor of SRMH in one of the two populations.

The results from the regression of weekly HED on gender demonstrated that First Nations and Métis females had lower odds of engaging in weekly HED than First Nations and Métis males. This was the result we expected based on the findings of Ryan et al.—who used the APS data in their study on heavy drinking—and it is in line with research on HED in the general population of Canada [2, 36, 45]. However, there are two important distinctions between our results and the conclusions of Ryan et al. Firstly, Ryan et al.’s study examined determinants of *heavy drinking*—defined by Statistics Canada as consuming five or more drinks, on one occasion, one or more times per month [2, 45]. In other words, heavy drinking refers to monthly HED, whereas our study was concerned with weekly versus non-weekly HED. Differences in the operationalization of HED are frequent in the literature, with studies examining everything from yearly to daily consumption of alcohol [2, 13–15]. Our results extend the findings of Ryan et al. (2016) to respondents who engaged in HED more frequently, a population that is potentially more vulnerable to depressive symptoms, as well as problems with alcohol abuse and associated suicidal ideation/attempts [15, 16, 42].

While Ryan et al. compared the odds of heavy drinking between First Nations and Métis, they did not test if gender was a significant predictor of heavy drinking in each of the separate Indigenous populations [2]. Our results suggest that gender was not a significant predictor of HED among the Inuit population of Canada. Similarly, while the relationship between gender and SRMH among Inuit respondents went in the expected direction, the relationship was not significant. It is possible that these null findings among Inuit respondents represent a real difference between the three Indigenous groups, although more likely they are attributable to aforementioned limitations in the size of the Inuit sample. Stratifying our analysis by both Indigenous identity and gender allowed us to more properly account for differences in socioeconomic status, environments, history, and culture among the different Indigenous populations [1, 37]. Although this stratification did not reveal substantial differences between the three groups, it allows for the results of our analysis to be generalized between the different populations with more confidence.

### Social capital and mental health outcomes

According to the results from the regression of weekly HED, high levels of Indigenous-specific social capital are significantly associated with lower odds of weekly HED, but only when examining all Indigenous or only First Nations men. In the literature, substance use among Indigenous populations has been linked to colonialism and the damages it caused to Indigenous cultures and communities [46, 47]. Reconnection to these communities and cultures is proposed as a possible protective factor against—and cure for—alcohol related issues, as well as an indicator of resilience among Indigenous populations [2, 46, 47]. Our results suggest that the relationship between Indigenous-specific sources of social capital—plus the protective effects they may provide—and HED is more important for men than women. In addition, this relationship does not appear to be important in predicting, or protecting against, HED among Métis men. Given that Ryan et al., found that Métis identity did not significantly affect the odds of heavy drinking, this again illustrates the importance of treating the distinct Indigenous populations in Canada as separate groups analytically.

Among the Inuit population, high levels of Indigenous specific social capital were significantly associated with lower odds of weekly HED among Inuit men, but only when *family ties* were included in the model. Meanwhile, among Inuit women high levels of Indigenous specific social capital were associated with much *higher* odds of reporting weekly HED. However, these results should be interpreted with caution due to the small cell size and large confidence intervals. These surprising findings could also be due to the unique geographic, demographic, and socioeconomic characteristics of the Inuit population in Canada. The 2011 National Household Survey—from which the sample population of the APS is derived—found that 73% of Inuit respondents lived in Inuit Nunangat—regarded as the original territory or homeland of the Inuit people of Canada [48]. Inside Nunangat, there are marked regional variations in alcohol consumption and social capital [48]. Depending on what regions were most represented in the relatively small analytic Inuit sample, the results could be very different. Therefore, instead of generalizing to the entire Inuit population, these results should be taken as a basis for future, more in depth research into the determinants of HED among the Inuit population of Canada.

Overall, the strength of family ties was not significantly associated with HED, expect among First Nations women. However, this variable only captures one aspect of what might be included in a typical measure of social capital. For example, someone could have strong ties to neighbors or other community members, but not their family members. Furthermore, the contradictory nature of the literature on social capital and alcohol consumption makes it difficult to compare First Nations women to other populations [25–27].

The models examining SRMH as the outcome provided contrasting results. Strong family ties in the community were significantly associated with lower odds of reporting fair/poor SRMH in every single sample population except for the Inuit, whereas Indigenous-specific social capital was not significant in any population. The fact that Indigenous-specific social capital was associated with the odds of weekly HED among men but not the odds of fair/poor SRMH among women does not fit with our initial hypothesis. Based on prior literature, we predicted that male respondents would report higher odds of weekly HED, and we would therefore expect a stronger association between Indigenous-specific social capital and HED among men—which was the case. However, Indigenous-specific social capital was not significantly associated with the odds of reporting fair/poor SRMH among women, even though there was a significantly higher prevalence of fair/poor SRMH among female respondents. These contradicting results raise questions about why Indigenous-specific social capital appeared to protect against weekly HED, but not fair/poor SRMH.

One explanation, based on our original hypothesis, could be that this particular measure of Indigenous-specific social capital is a more salient predictor of health outcomes among men, and it is therefore more strongly associated with the health outcome that was more prevalent among men. There is some evidence in the literature that women have higher average levels of social support than men, due in part to access to broader social networks outside the family [49]. Furthermore, the stressful components of these extrafamilial social networks have been shown to be more strongly associated with mental health than the positive components [49]. In this case the Indigenous-specific social capital variable mostly encompassed extrafamilial social networks, which could explain why this variable was not significant among female respondents.

The other possibility is that the Indigenous-specific social capital measure is simply a better predictor of weekly HED overall, and strength of family ties is a better predictor of SRMH. Strength of family ties could represent something besides social capital—perhaps familial support—that is a more salient predictor of SRMH. Another possibility is that these two variables genuinely capture different types of social capital. In regard to respondents’ perception of their mental health, immediate connections to social networks through family are potentially more important. Meanwhile, the odds of engaging in HED, a harmful behavior, are lower among those deeply ingrained in the social networks of their communities. This explanation would fit with prior research that links discrete dimensions of social capital—such as social participation or structural social capital—to specific health outcomes [19–21]. It might be worthwhile to perform a more robust comparison of Indigenous-specific social capital and the different dimensions of social capital—using a survey specifically designed to address this question—in order to gain further understanding into the role of social capital on the mental health of Indigenous populations.

### Limitations and next steps

One of the limitations of using the 2012 cycle of the APS is that the sample is restricted to off-reserve Indigenous populations. On-reserve Indigenous populations may be exposed to different physical environments or systemic barriers, and have different cultural and historical experiences than off-reserve Indigenous populations. For example, Skinner et al. found that the prevalence of food insecurity among on-reserve First Nations population in Ontario was more than double the prevalence in off-reserve Indigenous households [50]. Off-reserve Indigenous populations are also less likely to speak an Indigenous language or to have attended a residential school system [51]. Therefore, social capital could play a very different role among on-reserve Indigenous populations. A future avenue of research could be to examine the relationship between social capital and mental health outcomes among on-reserve populations using the *First Nations Regional Health Survey* (FNHRS)—a First Nations governed national health survey that specifically focuses on northern and on-reserve First Nations populations in Canada [52].

The results of this paper are also limited by the self-reported nature of the dependent variables—self-rated mental health and the self-reported frequency of consuming five or more drinks in one sitting. Alcohol consumption is particularly vulnerable to under reporting, due to both recall and social desirability bias. Those who drink frequently or heavily are more likely to under report their drinking, and respondents typically report median rather than mean alcohol consumption [53, 54]. Our analysis was further limited by the upper age limit—45 years old—imposed by several of the social capital variables. While it ensures a more homogenous sample with regards to the historical context of Indigeneity, older populations may have different experiences with Indigenous-specific social capital. For example, the percentage of Indigenous people who speak an Indigenous language varies greatly with age [51], and thus our results only apply to younger generations. There is also a recognizable risk of reverse causality in our analysis of social capital as a predictor of HED and SRMH. Our social capital variable may have mitigated this problem by capturing exposure to Indigenous networks and communities in childhood, but retrospective self-reports are liable to be influenced by respondents’ current situation.

Finally, since the results from this paper were based on male/female identities they cannot be generalized to all Indigenous populations, such as those who identify as *two-spirit*—a term traditionally used by certain Indigenous groups to indicate a gender identity different from the typical conceptions of male and female [55]. However, we could not find any studies indicating the percentage of the Indigenous population of Canada that identify as *two-spirit,* so it is unknown how substantially this would affect the generalizability of results.

## Conclusions

Using data from the 2012 cycle of the Aboriginal Peoples Survey, we investigated gender variations in the relationship between social capital and two mental health outcomes—self-rated mental health and heavy episodic drinking—among the Indigenous populations of Canada. Our analysis revealed significant gender differences in the odds of reporting fair/poor SRMH and HED. We also found evidence of a relationship between these health outcomes and different types of social capital, although the strength and direction of this relationship varied across all three Indigenous populations. Beyond addressing a critical gap in the literature on gender differences in the odds of reporting fair/poor SRMH and HED, our results also support further investigation into the role that social capital and particularly Indigenous-specific forms of social capital may play as a determinant of health among different Indigenous populations.

## Data Availability

The dataset analyzed for this study is not available for public dissemination due to the privacy restrictions set out by the Federal Government of Canada regarding the collection and safeguarding of census/survey data. However, the data can be accessed through an application to Statistics Canada through the Canadian Research Data Centre Network.

## Appendix

**Table 9:** Logistic regression for weekly heavy episodic drinking regressed only on social capital—separated by identity and gender

**Total Sample Population**		
	Odds Ratio for Males (Confidence Interval)	Odds Ratio for Women (Confidence Interval)
Indigenous-Specific Social Capital		
Low-Levels	–	–
High-Levels	0.68 (0.51–0.90)*	0.92 (0.65–1.31)
**First Nations**		
	Odds Ratio for Males (Confidence Interval)	Odds Ratio for Women (Confidence Interval)
Indigenous-Specific Social Capital		
Low-Levels	–	–
High-Levels	0.54 (0.36–0.81)*	0.70 (0.42–1.16)
**Métis**		
	Odds Ratio for Males (Confidence Interval)	Odds Ratio for Women (Confidence Interval)
Indigenous-Specific Social Capital		
Low-Levels	–	–
High-Levels	0.96 (0.59–1.57)	0.89 (0.47–1.69)
**Inuit**		
	Odds Ratio for Males (Confidence Interval)	Odds Ratio for Women (Confidence Interval)
Indigenous-Specific Social Capital		
Low-Levels	–	–
High-Levels	0.36 (0.13–1.01)	2.91 (1.05–8.06)*

95% confidence intervals; **p* < 0.05; adjusting for age, education, income, and urban/rural location

## Abbreviations

APS: Aboriginal Peoples Survey
HED: Heavy episodic drinking
SRMH: Self-rated mental health

## References

[1] Nelson SE, Wilson K (2017). The mental health of indigenous peoples in Canada: a critical review of research. Soc Sci Med.

[2] Ryan CJ, Cooke M, Leatherdale ST (2016). Factors associated with heavy drinking among off-reserve first nations and Métis youth and adults: evidence from the 2012 Canadian aboriginal peoples survey. Prev Med.

[3] Lawrence B (2003). Gender, race, and the regulation of native identity in Canada and the United States: an overview. Hypatia.

[4] de Leeuw S, Maurice S, Holyk T, Greenwood M, Adam W (2012). With reserves: colonial geographies and first nations health. Ann Assoc Am Geogr.

[5] Ahmad F, Jhajj AK, Stewart DE, Burghardt M, Bierman AS (2014). Single item measures of self-rated mental health: a scoping review. BMC Health Serv Res.

[6] Browne AJ (2007). Clinical encounters between nurses and first nations women in a Western Canadian hospital. Soc Sci Med.

[7] Robertson LH. The residential school experience: syndrome or historic trauma. Pimatisiwin. 2006;4(1):1–28.

[8] Tang SY, Browne AJ (2008). ‘Race’matters: racialization and egalitarian discourses involving aboriginal people in the Canadian health care context. Ethn Health.

[9] Mawani FN, Gilmour H (2010). Validation of self-rated mental health. Health Rep.

[10] Bougie E, Arim RG, Kohen DE, Findlay LC (2016). Validation of the 10-item Kessler psychological distress scale (K10) in the 2012 aboriginal peoples survey. Health Rep.

[11] Corna LM, Cairney J, Streiner DL (2010). Suicide ideation in older adults: relationship to mental health problems and service use. The Gerontologist.

[12] Ahenakew C (2011). The birth of the ‘Windigo’: the construction of aboriginal health in biomedical and traditional indigenous models of medicine. Crit Lit Theor Pract.

[13] Elton-Marshall T, Leatherdale ST, Burkhalter R (2011). Tobacco, alcohol and illicit drug use among aboriginal youth living off-reserve: results from the youth smoking survey. Can Med Assoc J.

[14] Mason-Jones AJ, Cabieses B (2015). Alcohol, binge drinking and associated mental health problems in young urban Chileans. PLoS One.

[15] Choi NG, DiNitto DM (2011). Heavy/binge drinking and depressive symptoms in older adults: gender differences. Int J Geriatr Psychiatry.

[16] Sung Y-k, La Flair LN, Mojtabai R, Lee L-C, Spivak S, Crum RM (2016). The Association of Alcohol use Disorders with suicidal ideation and suicide attempts in a population-based sample with mood symptoms. Arch Suicide Res.

[17] Hunter B (2000). Social exclusion, social capital, and indigenous Australians: measuring the social costs of unemployment.

[18] Frank C, Davis CG, Elgar FJ (2014). Financial strain, social capital, and perceived health during economic recession: a longitudinal survey in rural Canada. Anxiety Stress Coping.

[19] Fujiwara T, Kawachi I (2008). Social capital and health: a study of adult twins in the U.S. Am J Prev Med.

[20] Hamano T, Fujisawa Y, Ishida Y, Subramanian S, Kawachi I, Shiwaku K (2010). Social capital and mental health in Japan: a multilevel analysis. PLoS One.

[21] Nieminen T, Martelin T, Koskinen S, Aro H, Alanen E, Hyyppä MT (2010). Social capital as a determinant of self-rated health and psychological well-being. Int J Public Health.

[22] Åslund C, Nilsson KW (2013). Social capital in relation to alcohol consumption, smoking, and illicit drug use among adolescents: a cross-sectional study in Sweden. Int J Equity Health.

[23] Kawachi I (2006). Social capital and community effects on population and individual health. Ann N Y Acad Sci.

[24] Weitzman ER, Chen Y-Y (2005). Risk modifying effect of social capital on measures of heavy alcohol consumption, alcohol abuse, harms, and secondhand effects: national survey findings. J Epidemiol Community Health.

[25] Lindström M (2005). Social capital, the miniaturization of community and high alcohol consumption: a population-based study. Alcohol Alcohol.

[26] Nieminen T, Prättälä R, Martelin T, Härkänen T, Hyyppä MT, Alanen E, Koskinen S (2013). Social capital, health behaviours and health: a population-based associational study. BMC Public Health.

[27] Greiner KA, Li C, Kawachi I, Hunt DC, Ahluwalia JS (2004). The relationships of social participation and community ratings to health and health behaviors in areas with high and low population density. Soc Sci Med.

[28] Brave Heart MYH, Chase J, Elkins J, Altschul DB (2011). Historical trauma among indigenous peoples of the Americas: concepts, research, and clinical considerations. J Psychoactive Drugs.

[29] Whitbeck BL, Chen X, Hoyt DR, Adams GW (2004). Discrimination, historical loss and enculturation: culturally specific risk and resiliency factors for alcohol abuse among American Indians. J Stud Alcohol.

[30] Phillips SP (2005). Defining and measuring gender: a social determinant of health whose time has come. Int J Equity Health.

[31] Read J n G, Gorman BK (2010). Gender and health inequality. Annu Rev Sociol.

[32] WHO (1998). Gender and health: technical paper.

[33] Beauboeuf-Lafontant T (2007). You have to show strength: an exploration of gender, race, and depression. Gend Soc.

[34] Ball J (2009). Fathering in the shadows: indigenous fathers and Canada's colonial legacies. Ann Am Acad Pol Soc Sci.

[35] Firestone M, Tyndall M, Fischer B (2015). Substance use and related harms among aboriginal people in Canada: a comprehensive review. J Health Care Poor Underserved.

[36] Matheson Flora I, White Heather L, Moineddin Rahim, Dunn James R, Glazier Richard H (2011). Drinking in context: the influence of gender and neighbourhood deprivation on alcohol consumption. Journal of Epidemiology and Community Health.

[37] O'Donnell V, Wallace S (2011). First Nations, Métis and Inuit women. Women in Canada: A gender-based statistical report.

[38] CIHR Indigenous Health Research. Action Plan: Building a healthier future for First Nations, Inuit and Métis people. http://www.cihr-irsc.gc.ca/e/50372.html. Accessed 4 Aug 2018.

[39] CIHR; NSERC; SSHRC (2014). Chapter Nine: Research Involving the First Nations, Inuit and Métis Peoples of Canada. Tri-Council Policy Statement: Ethical Conduct for Research Involving Humans.

[40] IJIH Defining Aboriginal People Within Canada. https://journals.uvic.ca/journalinfo/ijih/IJIHDefiningIndigenousPeoplesWithinCanada.pdf. Accessed 10 May 2018.

[41] Lin E, Chan B, Goering P (1998). Variations in mental health needs and fee-for-service reimbursement for physicians in Ontario. Psychiatr Serv.

[42] Fillmore MT, Jude R (2011). Defining “binge” drinking as five drinks per occasion or drinking to a 0.08% BAC: which is more sensitive to risk?. Am J Addict/Am Acad Psychiatr Alcohol Addict.

[43] Milloy JS. A national crime: The Canadian government and the residential school system, vol. 11. Winnipeg: Univ. of Manitoba Press; 2017.

[44] Hallett D, Chandler MJ, Lalonde CE (2007). Aboriginal language knowledge and youth suicide. Cogn Dev.

[45] Canada, S. Heavy Drinking, 2012. https://www150.statcan.gc.ca/n1/pub/82-625-x/2013001/article/11838-eng.htm. Accessed 18 Dec 2018.

[46] Duran E. Healing the soul wound: counseling with American Indians and other native people. New York: Teachers College Press; 2006.

[47] Whitbeck LB, Adams GW, Hoyt DR, Chen X (2004). Conceptualizing and measuring historical trauma among American Indian people. Am J Community Psychol.

[48] Wallace SE (2014). Inuit health: selected findings from the 2012 aboriginal peoples survey.

[49] Kawachi I, Berkman LF (2001). Social ties and mental health. J Urban Health.

[50] Skinner K, Hanning RM, Tsuji LJ (2014). Prevalence and severity of household food insecurity of first nations people living in an on-reserve, sub-Arctic community within the Mushkegowuk territory. Public Health Nutr.

[51] Reading CL, Wien F (2009). Health inequalities and the social determinants of aboriginal peoples' health.

[52] FNIGC. About RHS. https://fnigc.ca/our-work/regional-health-survey/about-rhs.html. Accessed 15 Dec 2018.

[53] Boniface S, Kneale J, Shelton N (2014). Drinking pattern is more strongly associated with under-reporting of alcohol consumption than socio-demographic factors: evidence from a mixed-methods study. BMC Public Health.

[54] Stockwell T, Donath S, Cooper-Stanbury M, Chikritzhs T, Catalano P, Mateo C (2004). Under-reporting of alcohol consumption in household surveys: a comparison of quantity–frequency, graduated–frequency and recent recall. Addiction.

[55] Mayo J, Sheppard M (2012). New social learning from two spirit native Americans. J Soc Stud Res.

